# Repeated prefrontal tDCS for improving mental health and cognitive deficits in multiple sclerosis: a randomized, double-blind, parallel-group study

**DOI:** 10.1186/s12967-024-05638-1

**Published:** 2024-09-13

**Authors:** Nasim Zakibakhsh, Sajjad Basharpoor, Hamidreza Ghalyanchi Langroodi, Mohammad Narimani, Michael A Nitsche, Mohammad Ali Salehinejad

**Affiliations:** 1 Department of Psychology, Faculty of Educational Sciences and Psychology, University of Mohaghegh Ardabili, Ardabil, Iran; 2 Clinical Development and Research Unit, Ghaem Hospital, Guilan University of Medical Sciences, Rasht, Iran; 3 Department of Psychology and Neurosciences, Leibniz Research Centre for Working Environment and Human Factors, Dortmund, Germany; 4 Bielefeld University, University Hospital OWL, Protestant Hospital of Bethel Foundation, University Clinic of Psychiatry and Psychotherapy, Bielefeld, Germany; 5 German Center for Mental Health (DZPG), Bochum, Germany; 6 School of Cognitive Sciences, Institute for Research in Fundamental Sciences, Tehran, Iran

**Keywords:** Multiple sclerosis, Transcranial direct current stimulation, Mental health, Cognitive deficits, Quality of life, Prefrontal tDCS

## Abstract

**Background:**

Multiple Sclerosis (MS) is an autoimmune disease associated with physical disability, psychological impairment, and cognitive dysfunctions. Consequently, the disease burden is substantial, and treatment choices are limited. In this randomized, double-blind study, we conducted repeated prefrontal electrical stimulation in 40 patients with MS to evaluate mental health variables (quality of life, sleep difficulties, psychological distress) and cognitive dysfunctions (psychomotor speed, working memory, attention/vigilance), marking it as the third largest sample size tDCS research conducted in MS to date.

**Methods:**

The patients were randomly assigned (block randomization method) to two groups of sham (*n* = 20), or 1.5-mA (*n* = 20) transcranial direct current stimulation (tDCS) targeting the left dorsolateral prefrontal cortex (F3) and right frontopolar cortex (Fp2) with anodal and cathodal stimulation respectively (electrode size: 25 cm^2^). The treatment included 10 sessions of 20 min of stimulation delivered every other day. Outcome measures were MS quality of life, sleep quality, psychological distress, and performance on a neuropsychological test battery dedicated to cognitive dysfunctions in MS (psychomotor speed, working memory, and attention). All outcome measures were evaluated at the pre-intervention and post-intervention assessments. Both patients and technicians delivering the stimulation were unaware of the type of stimulation being used.

**Results:**

Repeated prefrontal real tDCS significantly improved quality of life and reduced sleep difficulties and psychological distress compared to the sham group. It, furthermore, improved psychomotor speed, attention, and vigilance compared to the sham protocol. Improvement in mental health outcome variables and cognitive outperformance were interrelated and could predict each other.

**Conclusions:**

Repeated prefrontal and frontopolar tDCS ameliorates secondary clinical symptoms related to mental health and results in beneficial cognitive effects in patients with MS. These results support applying prefrontal tDCS in larger trials for improving mental health and cognitive dysfunctions in MS.

**Trial registration:**

ClinicalTrials.gov Identifier: NCT06401928.

**Supplementary Information:**

The online version contains supplementary material available at 10.1186/s12967-024-05638-1.

## Introduction

Multiple Sclerosis (MS) is the most common autoimmune disorder of the central nervous system, afflicting more than 2.5 million people worldwide, especially young people [1, 2]. It is a progressive chronic disease, caused by an autoimmune attack, which results in the gradual loss of the myelin sheath around neuronal axons of the central nervous system. Depending on the affected neurons, different symptoms are expressed in the course of the disease; nonetheless, physical disability, cognitive impairment, and decreased quality of life are common in MS [3]. MS results in motor, sensory, cognitive, and neuropsychiatric symptoms, all of which can occur independently of one another [4]. The disease is associated with a high mental health burden due to primary symptoms of sensory and motor deficits. Some of the most common symptoms include fatigue, vision problems, numbness and tingling, muscle spasms, stiffness and weakness, mobility problems, and pain [2, 5].

In addition to primary symptoms, individuals with MS also experience mental health challenges, affecting quality of life, sleep, and emotional disturbances [6–8]. Cognitive deficits are also commonly observed symptoms in MS and include deficits in attention and vigilance, information processing, executive functioning, processing speed, and long-term memory [4] with a profound effect on activities of daily living [9]. Both primary and secondary symptoms can vary in severity and may come and go, depending on disease activity, progression, and the type of MS, which include relapsing-remitting, or chronic-progressive types [8, 10]. Individuals with MS need to work closely with healthcare professionals to manage their symptoms and improve their quality of life. Considering the burden of treatment [11], the complex pathophysiology and psychophysiology, and limited treatment options for secondary symptoms (e.g., physical therapy, psychotherapy, cognitive rehabilitation) [3], there is a need for novel and effective treatment for both primary and secondary symptoms in MS.

Transcranial direct current stimulation (tDCS) is a safe and easy-to-use noninvasive brain stimulation (NIBS) intervention for studying and modifying human brain functions [12, 13] and immune-related neurotransmitters like dopamine [14, 15]. In mental health settings, tDCS has advantages over other NIBS methods, including affordability, fewer side effects, and suitability for home use and remote control. It involves applying a low-intensity direct current to the scalp, which induces alterations of the resting membrane potential of neurons. At the macroscale level, anodal stimulation depolarizes neurons at a subthreshold level, making them more likely to fire action potentials, while cathodal stimulation hyperpolarizes neurons, reducing their excitability [16, 17]. By modulating cortical excitability parameters (e.g., cortical inhibition and facilitation) and inducing neuroplasticity effects, it is possible to restore functional brain abnormalities and affect target behavior/cognition. Previous studies have shown functional brain abnormalities in MS that are also related to secondary symptoms in MS [18, 19]. These abnormalities can take many different forms, such as altered brain activity, neural network disturbances, and compromised cognitive functions. Key elements of functional brain impairments in MS include changes in pain processing pathways as well as alterations of brain regions involved in the regulation of mood and arousal, including the limbic system, hypothalamus, and motor regions [20–22]. Furthermore, there is growing evidence of grey matter changes in MS that are linked to disability and other clinical symptoms in these patients [23, 24].

Previous tDCS studies in MS have mostly focused on examining the efficacy of prefrontal and motor cortices stimulation on clinical symptoms (e.g., fatigue, pain), motor symptoms, and cognitive deficits [25–28]. There is inconsistency in the studies results regarding the cognitive effects of NIBS, including tDCS, on MS patients. In addition to cognitive and motor symptoms, MS patients often experience other mental health issues that are less frequently addressed in tDCS and NIBS studies. While there have been some studies on the effects of tDCS on mental health-related variables, such as quality of life [29], sleep [30], and emotional difficulties [31], there is still a lack of research on the impact of tDCS on both mental health-related variables and cognitive performance in patients with MS. Accordingly, the purpose of this randomized, sham-controlled study with a parallel-group design is to address this research gap by examining [1] the effects of repeated prefrontal-frontopolar tDCS on mental health variables (i.e., quality of life, sleep difficulties, psychological distress) in MS patients [2], the impact of repeated prefrontal-frontopolar tDCS on their cognitive performance, and [3] the association between these two sets of secondary symptoms.

## Methods

### Participants

**Fig. 1:**
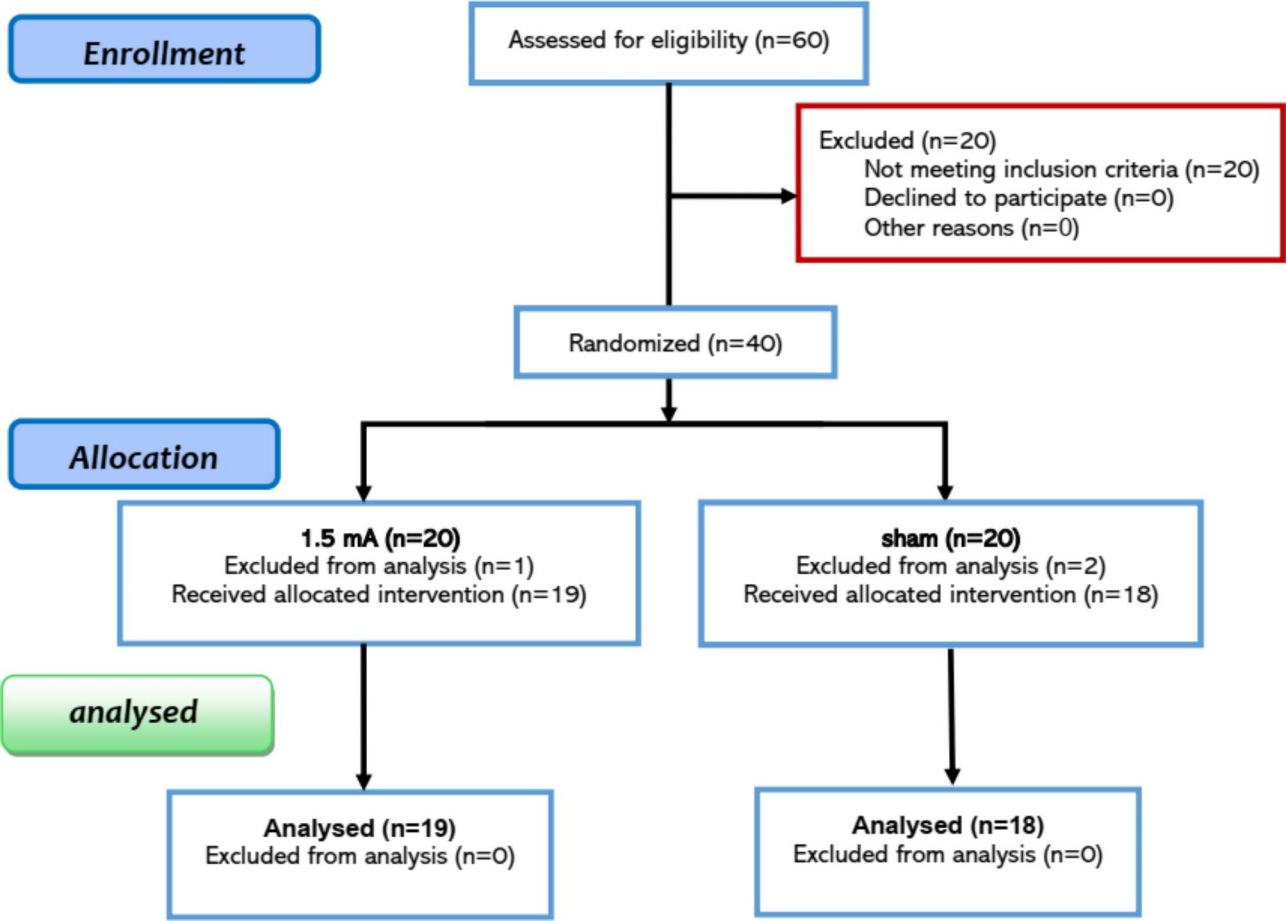
The CONSORT Flow Diagram outlining the study’s inclusion procedures. Thirty-seven patients completed all post-intervention CANTAB subtests, while five did not attend the mental health assessment

Eighty MS patients from the local MS community (Rasht, Iran) were screened for inclusion in the study. The inclusion criteria were: [1] diagnosis of MS according to the Diagnostic criteria for multiple sclerosis: 2010 Revisions of the McDonald criteria (Polman et al., 2011), certified by a professional neurologist [2], being 25–55 years old [3], being non-smoker [4], no previous history of neurological diseases, brain surgery, epilepsy, seizures, brain damage, head injury, or metal brain implants [5], absence of other psychiatric disorders except mood and anxiety disorders, and [6] no relapse of MS symptoms within the last two months before beginning the experiment. Of those who met the inclusion criteria (*n* = 60), forty patients were randomly assigned to the experimental (active tDCS) and control (sham tDCS) groups based on a sample size analysis (f = 0.30, α = 0.05, power = 0.95, mixed-model ANOVA for 2 groups with 2 measurements) which resulted in a sample size of 40 patients. This sample size is larger than 94% of tDCS studies reported in recent metaanalyss and review studies [26, 32]. For the group assignment of the participants, block randomization method was applied. Three patients decided to withdraw from the study following the first session and thus the final analysis was conducted on 37 patients (mean age = 37.30, SD = 6.21, 27 females, 10 males) (see Table 1; Fig. 1 for demographics and study inclusion). This is a retrospectively registered clinical trial (ClinicalTrials.gov Identifier: NCT06401928) approved by the Ethics Committee of the Mohaghegh Ardabili University (Ethics code: IR.MAU.REC.1401.94). Participants gave their written informed consent before participation.

**Table 1:** Demographic data

	Active tDCS	Sham tDCS	*p*-value*
Sample size (n)	19	18	
Age- M (SD)	36.89 (6.41)	37.72 (6.15)	0.691
Sex – Male (female)	4 (15)	6 (12)	0.401
Marital Status – Single (married)	9 (10)	7 (11)	0.603
OAID yes (No)	4 (15)	1 (17)	0.340
Family history- yes (No)	9 (10)	6 (12)	0.508
**Duration of disease**			0.078
1–10 years	14	7	
11–20 years	4	10	
More than 20 years	1	1	
**Type of MS**			0.285
CIS	3	5	
RR	14	13	
SR	2	0	
**Type of medications** IS (IM)	10 (7)	12 (5)	0.663

*Note* tDCS = transcranial Direct Current Stimulation; M = Mean; SD = standard deviation; OAID = other autoimmune disease; CIS = clinically isolated syndrome; RR = relapsing-remitting multiple sclerosis; SP = secondary progressive multiple sclerosis; IS = immunosuppressant drug; IM = immunomodulatory drug; * = between-group differences in demographic variables were explored by Chi-square tests or Fisher’s exact test for categorical variables and *F*-tests for continuous variables

### Measures

#### Mental health assessments

The primary outcome measures of the study included quality of life, sleep difficulties, and psychological distress that were measured with the Multiple Sclerosis Impact Scale (MSIS-29) [33], the Mini Sleep Questionnaire (MSQ) [34], and the Depression Anxiety Stress Scale-21 (DASS-21) [35] respectively. The MSIS-29 is a measure of the physical and psychological impact of MS from the patients’ perspective consisting of 29 questions with the first 20 items focusing on the physical impact and the remaining 9 on the psychological impact. The Mini Sleep Questionnaire (MSQ) is typically used to screen sleep disturbances in clinical populations, and the DASS-21 is a 21-item self-report measure designed to assess the severity of general psychological distress and symptoms related to depression, anxiety, and stress in adults and older adolescents (+ 17 years). Details about these measures and their psychometric properties can be found in the supplementary information. A native-language version of each test was used in this study [36–38].

#### Cognitive performance

In addition to mental health-related outcome variables, we assessed the neuropsychological performance and cognitive functioning of the MS patients with several subtests of a neuropsychological battery designed for MS patients using the Cambridge Cognition Neuropsychological Battery (CANTAB). The battery included 3 computerized tests to measure psychomotor speed, working memory and sustained attention/vigilance via the Reaction Time (RTI), Spatial Working Memory (SWM) and Rapid Visual Information Processing (RVP) tests respectively. The tests included in this battery are highly sensitive to cognitive impairments in MS across the disease severity spectrum [39]. Details about these measures are available in the supplementary information.

### tDCS

We used a two-channel Neurostim stimulator device (MadinaTeb, Tehran, Iran) powered by a 9-volt alkaline battery. Electrical current was applied through a pair of rubber electrodes placed in saline-soaked sponge (5×5 cm) for a period of 20 min (with 30 s ramping up and 30 s ramping down) and a stimulation intensity of 1.5 mA (current density of 0.06 mA/cm^2^). The intervention was conducted by a trained tDCS operator and impedance was monitored and kept below to ensure patients’ tolerability and consistent current flow in each session. We had two stimulation conditions: active and sham tDCS. In the active condition, the anodal electrode was placed over the left dorsolateral prefrontal cortex (left DLPFC) over the F3 electrode position according to the 10–20 EEG International System and the cathodal electrode was placed over the right frontopolar cortex (FPC) (electrode position Fp2) which includes orbitofrontal cortex (BA 10, 11) [40, 41], fixed with headbands. Both electrodes were positioned longitudinally along the medio-lateral axis of the target regions. To reduce shunting of current between the electrodes through the scalp, the edges had a distance of at least 6 cm. In the sham condition, a sham stimulation was employed with the same electrode configuration. Here, the electrical current was ramped up for 30 s followed by 30 s of stimulation and 30 s of ramping down to generate the same sensation as the active condition, and then was turned off without the participants’ knowledge. This method of sham stimulation has been proven reliable [42]. A survey was conducted after each session to document any reported side effects, but blinding efficacy (asking participants to guess about the type of stimulation) was not explored due to the multi-session design of the study. This aimed to prevent participants’ bias from habituation to tDCS-induced sensations over multiple sessions. After finalizing the study protocol, the patients in the sham group were assigned to active tDCS intervention, but the latter procedure was beyond the focus of the study protocol.

### Procedure

**Fig. 2:**
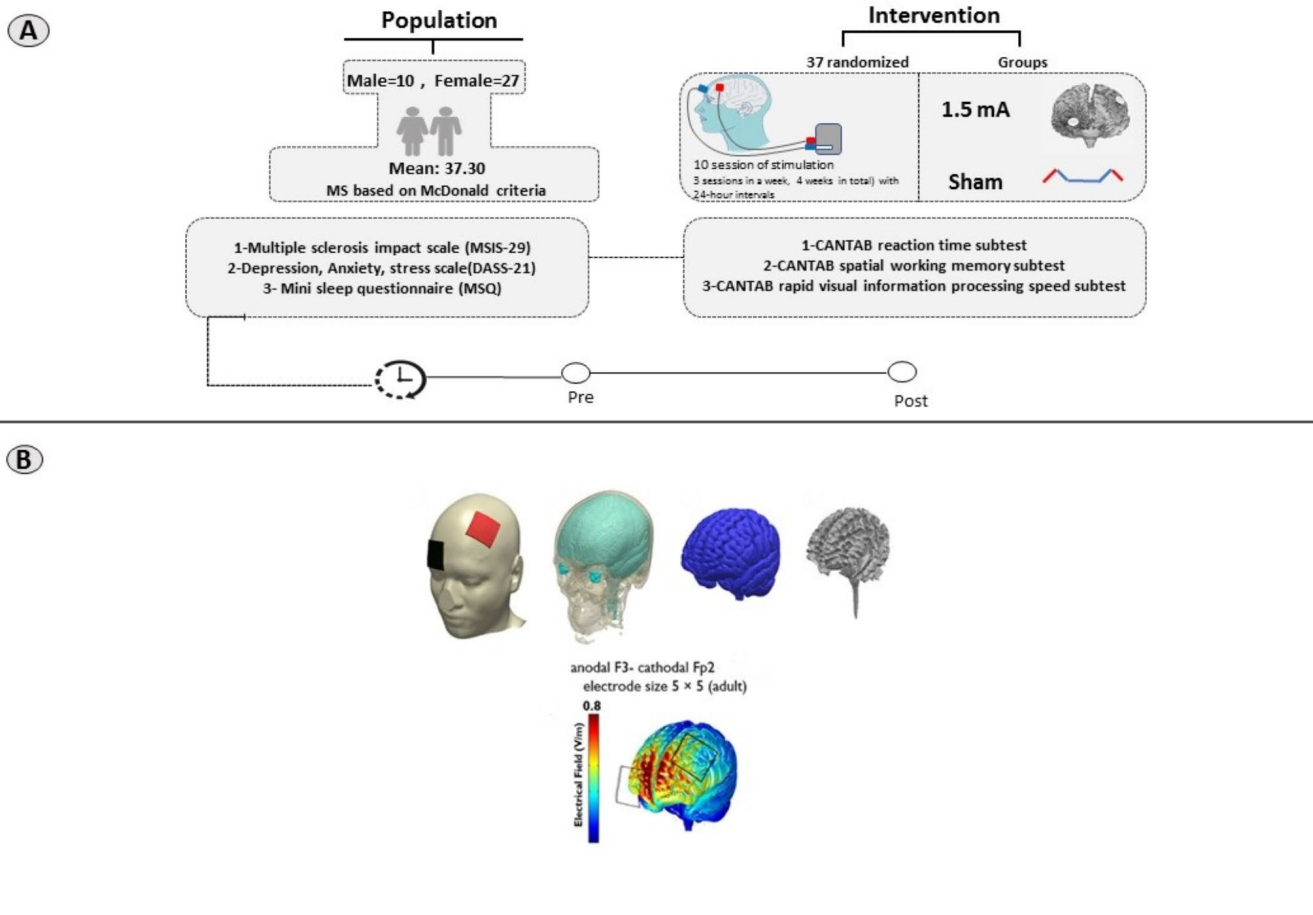
(**A**) This study was a randomized, double-blind trial. Participants were randomly assigned into two groups: active tDCS and sham tDCS. Both groups underwent pre- and post-intervention assessment of mental health-related variables and cognitive performance with the Cambridge Neuropsychological Test Automated Battery (CANTAB), MS battery. The anodal electrode was positioned over the left dorsolateral prefrontal cortex (DLPFC-F3), while the cathode was placed over the right frontopolar cortex (Fp2). (**B**) 3D models were utilized to examine the flow of electrical current in the brain following the specified protocol. The MR image was segmented into six tissue types: gray matter (GM), white matter (WM), CSF, skull, scalp, and air cavities using SPM8 from the Welcome Trust Center for Neuroimaging, London, UK, with an enhanced tissue probability map. The segmented images were used to create a 3D model with Simpleware software version 5 from Synopsys, Mountain View, CA, incorporating the electrodes and saline-soaked sponges. The distribution of current flow within the brain was then computed using the finite element method in COMSOL Multiphysics software version 5.2 from COMSOL Inc., Burlington, MA. The electric fields were visualized for stimulation intensities of 2.0 mA with an F3 anodal–Fp2 cathodal montage. *note*: This model illustrates the current flow for 2 mA tDCS for illustrative purposes. The induced electric field of 1.5 mA differs from the 2 mA field

Prior to the experiment, participants completed a brief questionnaire to evaluate their suitability for brain stimulation. Participants were instructed to prevent from caffein, nicotine, alcohol consumption and intensive physical activity before attending each stimulation session. The active tDCS and sham groups received 10 sessions of stimulation (three sessions per week, resulting in a total of three weeks plus an additional day) with 24-hour between-session intervals (except for the 4th and 8th sessions that had 48-h interval due to the weekend). Outcome measures (mental health and cognitive assessment) were evaluated before the first tDCS session (pre-intervention), and right after the end of the last tDCS session (post-intervention). All tDCS sessions were scheduled between 2:00 and 5:00 p.m. and patients were monitored for sleep pressure to mitigate a potential impact of circadian variation on cortical excitability and neuroplasticity induction for all participants across all sessions [43, 44]. All sessions were conducted in the MS clinical neuroscience private clinic (Rasht, Iran) in a dedicated space for research. Before starting the experiment, the participants were given instructions about the cognitive tasks. Each stimulation session took about 40 min (preparation, stimulation, wrapping up time). The measurement sessions before the intervention and after the intervention took about 2 h. Each session was conducted on two separate days to prevent fatigue. The first day was dedicated to clinical assessment (quality of life, sleep, psychological distress), and the second day for cognitive assessment (psychomotor speed, working memory, attention/vigilance) (see Fig. 2). Both participants and experimenter were blinded. To maintain a double-blind design, a separate investigator prepared the device, while a technician unaware of the stimulation type (sham vs. real) and group assignment administered the stimulation. An independent researcher, blind to stimulation conditions and group assignment, evaluated the outcome measures, performed data analysis, and determined group assignments.

### Statistical analysis

Data were analyzed with the statistical package SPSS, version 27.0 (IBM, SPSS, Inc., Chicago, IL). The normality and homogeneity of data variance were confirmed by Shapiro-Wilk and Levin tests, respectively. Mixed model ANOVAs were conducted for the dependent variables (MSIS, DASS, MSQ scores; psychomotor speed, working memory and attention tasks) with “group” (active vs. sham) as the between-subject and time (pre-intervention, post-intervention) as the within-subject factors. Mauchly’s test was used to evaluate sphericity of the data before performing the respective ANOVAs (*p* < 0.05). In case of violation, degrees of freedom were corrected using Greenhouse-Geisser estimates of sphericity. Post hoc comparisons were conducted with Fisher’s LSD post-hoc tests for individual mean difference comparisons across groups (active 1.5 mA, sham) and time points (pre-intervention, post-intervention). Pearson’s correlation and exploratory linear regression analyses were also calculated to explore potential associations between mental health-related variables and cognitive performance. Data analysis was conducted on all randomized patients who completed the pre-and post-intervention assessments without regard to adherence to their randomization assignment. The critical level of significance was 0.05 for all statistical analyses.

## Results

### Data overview

Demographic information is summarized in Table 1. Patients tolerated the stimulation well and no adverse effects were reported during and after stimulation. No significant difference was found between the group ratings of tDCS side effects except for the burning sensation (*p* = 0.044) which was higher in the active group (Table 2). The burning sensation, however, did not correlate with any outcome measure. No significant between-group differences were observed for baseline measurements of outcome variables except for mean latency of RVP (Table 3 All patients completed the post-intervention cognitive assessment (*n* = 37), but five did not finish the mental health assessment sessions (*n* = 32).

**Table 2:** Means and SDs of reported tDCS side effects

		active tDCS	Sham tDCS	t	df	*p*-value*
		M(SD)	M(SD)			
**Reported side effect**	Skin redness	0.32 (1.003)	0.11 (0.32)	0.84	21.88	0.408
	Sleep problem	0.32 (1.15)	0.0 (0.0)	1.18	18.00	0.25
	Fatigue	0.26 (1.14)	0.11 (0.47)	0.53	24.17	0.599
	Pain	0.21 (0.53)	0.17 (0.38)	0.28	32.64	0.775
	Burning	1.32 (1.63)	0.44 (0.705)	2.12	24.74	**0.044**
	Itching	0.84 (1.25)	0.56 (0.85)	0.81	31.82	0.422
	Tingling	1.05 (1.35)	1 (0.907)	0.14	31.60	0.890

Values are presented as means ± standard deviation (SD). Note: each value represents the average of side effects reported during all 10 tDCS sessions. tDCS = transcranial Direct Current Stimulation; M = Mean; SD = Standard Deviation; * = between-group differences of side effect items were explored by independent samples t-tests. Significant results are highlighted (*p* ≤ 0.05) in **bold**

**Table 3:** Means and SDs of outcome variables before and after the tDCS

Measure	Outcome variable	Time	active tDCS	sham tDCS	*p*-value
			M (SD)	M (SD)	
**quality of life (MSIS)**	Score	Pre-intervention	51.20 (19.19)	51.41 (13.94)	0.972
		Post-intervention	41.93 (11.74)	51.94 (16)	
**DASS**	Depression	Pre-intervention	3.73 (2.34)	6.11 (6.14)	0.168
		Post-intervention	2.06 (2.25)	6.76 (6.41)	
	Anxiety	Pre-intervention	3 (1.73)	3.64 (4.10)	0.575
		Post-intervention	1.60 (1.88)	4 (4.54)	
	Stress	Pre-intervention	6.73 (2.81)	7.58 (4.89)	0.575
		Post-intervention	4.53 (3.64)	8.29 (5.35)	
**Sleep**	Total score	Pre-intervention	13.20 (4.37)	11.29 (3.78)	0.197
		Post-intervention	7.26 (1.62)	11.58 (3.90)	
	Insomnia	Pre-intervention	6.46 (2.79)	5.17 (1.55)	0.112
		Post-intervention	3.46 (0.83)	5.35 (1.65)	
	Oversleep	Pre-intervention	6.73 (2.52)	6.11 (2.71)	0.513
		Post-intervention	3.80 (1.08)	6.23 (2.88)	
**RTI**	5-choice movement time	Pre-intervention	1491.37 (313.24)	1384.99 (448.08)	0.406
		Post-intervention	825.54 (294.49)	1294.09 (404.63)	
	5-choice reaction time	Pre-intervention	967.30 (415.51)	987.08 (346.31)	0.876
		Post-intervention	779.37 (392.55)	1037.36 (338.37)	
	Simple movement time	Pre-intervention	711.15 (208.95)	626.94 (226.50)	0.247
		Post-intervention	448.67 (157.16)	638.68 (191.55)	
	Simple reaction time	Pre-intervention	457.59 (101.29)	428.05 (112.48)	0.406
		Post-intervention	273.69 (86.51)	450.4 (102.97)	
**SWM**	Strategy	Pre-intervention	31.947 (5.83)	32.44 (5.90)	0.798
		Post-intervention	30.526 (6.73)	33.27 (5.53)	
	Total error	Pre-intervention	28.84 (23.59)	25.11 (24.83)	0.642
		Post-intervention	15.47 (12.23)	19.94 (10.87)	
**RVP**	Hits	Pre-intervention	14.947 (4.08)	15.27 (4.05)	0.807
		Post-intervention	18.736 (3.61)	14.38 (4.13)	
	Mean latency	Pre-intervention	837.90 (88.39)	470.72 (127.81)	< 0.001
		Post-intervention	538.33 (114.37)	480.73 (149.82)	

*Note* tDCS = transcranial Direct Current Stimulation; M = Mean; SD = Standard Deviation; MSIS = Multiple Sclerosis Impact Scale (quality of life); DASS = Depression, Anxiety, Stress Scale; MSQ = Mini Sleep Questionnaire; RTI = Reaction time test; SWM = Spatial Working Memory; RVP = Visual Information Processing; *p* values refer to baseline (pre-intervention) measurement comparisons using ANOVA tests

### Efficacy of tDCS on quality of life, sleep quality, and psychological distress

**Fig. 3:**
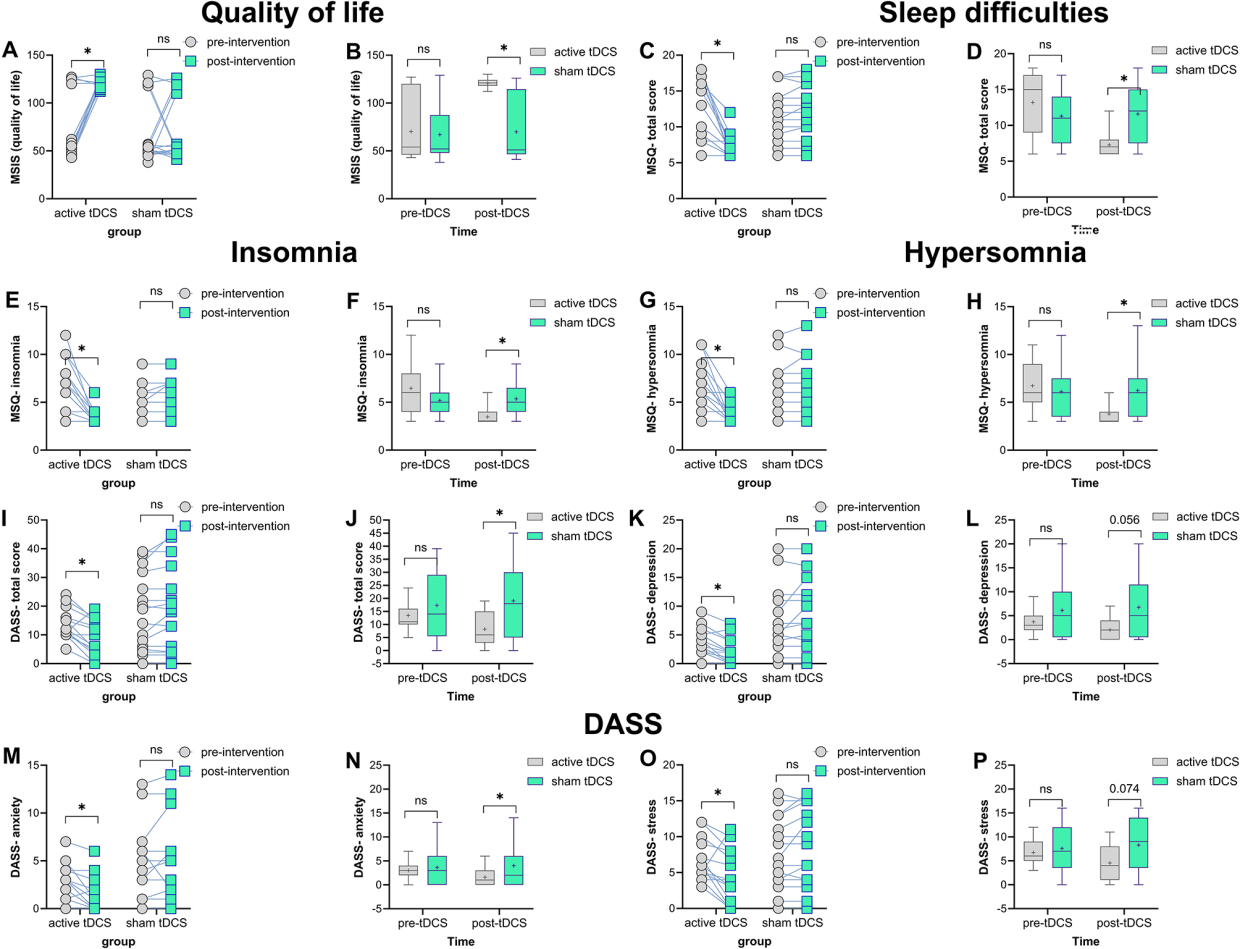
Effects of prefrontal active and sham tDCS on mental health variables. The left panel shows average outcome measures before and after the intervention for each group (within-group comparisons), while the right panels depict average measures at each time point (pre- and post-intervention) across groups (active vs. sham). *Note*: tDCS = transcranial Direct Current Stimulation; DASS = Depression Anxiety Stress Scale. Asterisks [*] in the left panels indicate significant differences between pre- and post-intervention measures in all groups. Asterisks in the right panels signify significant differences between active (1.5 mA) and sham tDCS at each time point. “ns” indicates non-significant results. All pairwise comparisons used Fisher’s LSD multiple comparisons tests, and error bars represent standard error of the mean (s.e.m.)

The results of the 2 (group) × 2 (time: pre, post) mixed model ANOVA showed a significant main effect of time (*F*_*1*_ = 7.94, *p* = 0.008, *η*p2 = 0.21) and a significant group×time interaction (*F*_*1*_ = 9.98, *p* = 0.004, *η*p2 = 0.25) but no main effect of group (*F*_*1*_ = 0.94, *p* = 0.338) on quality of life scores measured by the MSIS-29. Fisher’s LSD post-hoc tests showed a significant increase in quality of life scores in patients with MS who received active tDCS after the intervention compared to pre-intervention (*t* = 4.74, *p* < 0.001). When compared to the sham group, active tDCS significantly improved *quality of life* scores after the intervention (*t* = 4,93, *p* < 0.001). Baseline between-group comparisons (active groups vs. sham) showed no significant differences in the pre-intervention scores (Fig. 3A, B, Table S1).

With respect to the sleep quality scores measured by the MSQ, the results of a 2 × 2 mixed model ANOVA showed a significant main effect of time (*F*_*1,30*_=30.598, *p* < 0.001, *η*p2 = 0.505) and a significant group×time interaction (*F*_*1,30*_=37.314, *p* < 0.001, *η*p2 = 0.554) but no main effect of group (*F*_*1,30*_=1.065, *p* = 0.31). Fisher’s LSD post-hoc tests showed a significant improvement of sleep quality scores only in patients with MS that received active tDCS after the intervention compared to pre-intervention (*t*_*total sleep*_ =4.50, *p* < 0.001, *t*_*insomnia*_ =4.48, *p* < 0.001, *t*_*hypersomnia*_ =3.29, *p* < 0.001) but not in the sham group. When compared to the sham group, the active tDCS group significantly improved with respect to the sleep total score (*t* = 3.38, *p* = 0.001), insomnia (*t* = 2.90, *p* = 0.005) and hypersomnia (*t* = 2.82, *p* = 0.006). Baseline between-group comparisons were not significant (*p* > 0.05) (Fig. 3C-H).

For psychological distress, a 3 (domain: depression, anxiety, stress) × 2 (time: pre, post) × 2 (group) mixed model ANOVA was conducted. The results showed significant main effects of time (*F*_*1,30*_=6.13, *p* = 0.019, *η*p2 = 0.17), domain (*F*_*2,60*_=15.608, *p* < 0.001, *η*p2 = 0.342) and a significant group×time interaction (*F*_*1,30*_=23.505, *p* < 0.001, *η*p2 = 0.439). The main effect of group, and the domain×time, domain×group and domain×group×time interactions were not significant (Table S1). Fisher’s LSD post-hoc tests showed a significant decrease in the DASS total score only in patients with MS who received active tDCS after the intervention compared to pre-intervention (*t* = 2.46, *p* = 0.020) but not in the sham group. Compared to the sham group, the active tDCS group showed significantly reduced psychological distress (*t*_*depression*_ =2.73, *p* = 0.008, *t*_*anxiety*_ =1.99, df = 30, *p* = 0.050, *t*_*stress*_ =2.43, *p* = 0.017) after the intervention. Baseline between-group comparisons (active groups vs. sham) showed no significant differences in the pre-intervention scores (*p* > 0.05) (Fig. 3M-P).

### Efficacy of tDCS on cognitive functions in MS patients

#### Psychomotor speed

**Fig. 4:**
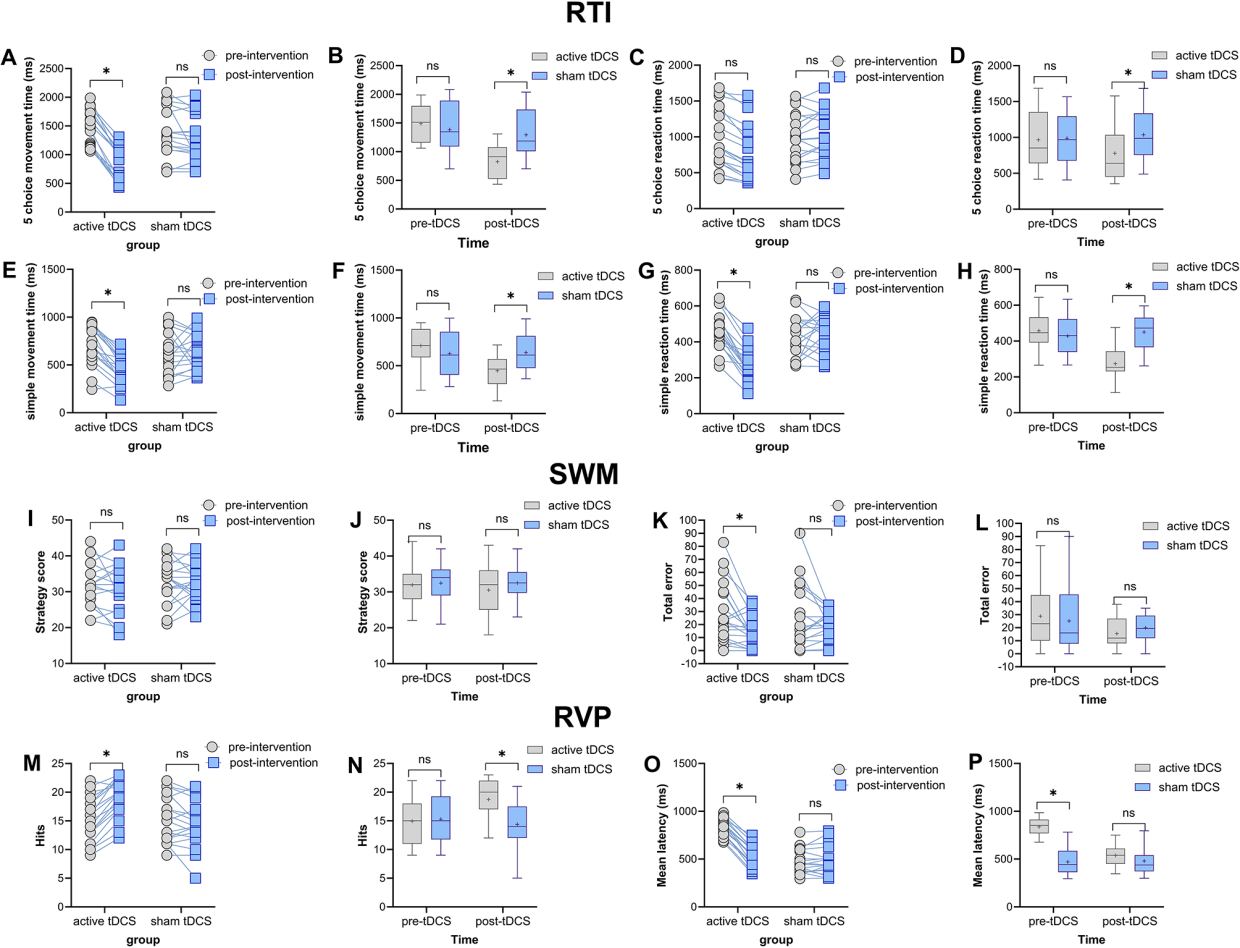
Effects of prefrontal active and sham tDCS on cognition. The left panel shows average outcome measures before and after the intervention for each group (within-group comparisons). The right panels present average measures at each time point (pre- and post-intervention) across groups (active vs. sham). *Note*: tDCS = transcranial Direct Current Stimulation; RTI = Reaction Time; SWM = Spatial Working Memory; RVP = Rapid Visual Processing. Floating asterisks [*] in the left panel indicate significant differences between pre- and post-intervention measurements in all groups. Asterisks in the right panel indicate significant differences between active stimulation (1.5 mA) and sham tDCS at each time point. “ns” denotes non-significant results. All pairwise comparisons used Fisher’s LSD multiple comparisons test, and error bars represent standard error of the mean (s.e.m.)

Psychomotor speed was evaluated with the RTI task. A significant main effect of time (*F*_*1,35*_=127.32, *p* < 0.001, *η*p2 = 0.784) and a significant group×time interaction (*F*_*1,35*_=73.491, *p* < 0.001, *η*p2 = 0.677), but no main effect of group was found for the 5-choice movement time. A similar significant main effect of time (*F*_*1,35*_=11.684, *p* = 0.002, *η*p2 = 0.25) and a significant group×time interaction (*F*_*1,35*_=34.992, *p* < 0.001, *η*p2 = 0.50), but a non-significant main effect of group was found for the 5-choice reaction time (Table S1). Fisher’s LSD post-hoc tests revealed a significantly faster movement time in patients who received active tDCS after the intervention compared to pre-intervention (*t* = 5.56, *p* < 0.001). Additionally, in the post-intervention condition, movement time was significantly shorter in the active group compared to the sham tDCS group (*t* = 3.86, *p* < 0.001). For reaction time, neither group responded faster after intervention as compared to the pre-intervention condition. However, performance after intervention was significantly faster in the active vs. sham group (*t* = 2.08, *p* = 0.043). Baseline between-group comparisons were not significant in both outcome variables (*p* > 0.05) (Fig. 4A-H).

Other outcome variables of interest in the RTI task were *simple*-choice movement time and reaction time. Here, the results of the mixed model ANOVA revealed a significant main effect of time (*F*_*1,35*_=17.476, *p* < 0.001, *η*p2 = 0.333) and a significant group×time interaction (*F*_*1,35*_=20.905, *p* < 0.001, *η*p2 = 0.374) but no main effect of group on movement time, and significant main effects of time (*F*_*1,35*_=24.729, *p* < 0.001, *η*p2 = 0.414) and group (*F*_*1,35*_=6.439, *p* = 0.016, *η*p2 = 0.155) and interaction between both variables (*F*_*1,35*_=40.317, *p* < 0.001, *η*p2 = 0.535) for the reaction time. Fisher’s LSD post-hoc tests showed significantly faster movement and reaction times in the psychomotor speed task in patients who received active tDCS after the intervention compared to pre-intervention (*t* = 4.09, *p* < 0.001; *t* = 5.60, *p* < 0.001). Both movement and reaction times in the psychomotor speed task after intervention were significantly faster in the active tDCS compared to the sham group (*t* = 2.92, *p* = 0.004; *t* = 5.31, *p* < 0.001). Baseline between-group comparisons were not significant for both outcome variables (*p* > 0.05) (Fig. 4A-H).

#### Working memory

Here strategy scores and total errors were analyzed. For the strategy scores, the results of the mixed model ANOVA showed no significant main effects of time, group, or their interaction (Table S1). For the total errors, however, a significant main effect of time (*F*_*1,35*_=9.081, *p* = 0.005, *η*p2 = 0.206) but no main effect of group or the group×time interaction was observed. Accordingly, no post hoc tests were conducted for errors (Fig. 4- I-L).

#### Attention and vigilance

The results of the mixed model ANOVA showed a significant main effect of time (*F*_*1,35*_=17.97, *p* < 0.001, *η*p2 = 0.337) and a significant group×time interaction (*F*_*1,35*_=46.299, *p* < 0.001, *η*p2 = 0.569) but no main effect of group (*F*_*1,35*_=2.53, *p* = 0.120) on hits (accurate responses). Patients who received active tDCS had a significantly higher number of hits after intervention vs. pre-intervention (*t* = 2.96, *p* = 0.004), and the number of hits was also significantly higher in the real intervention than the number of hits in those who received sham tDCS after intervention (*t* = 2.79, *p* = 0.006). Similarly, significant main effects of time (*F*_*1,35*_=211.259, *p* < 0.001, *η*p2 = 0.858), group (*F*_*1,35*_=30.05, *p* < 0.001, *η*p2 = 0.462) and group×time interaction (*F*_*1,35*_=241.50, *p* < 0.001, *η*p2 = 0.873) emerged for the mean reaction time. Fisher’s LSD post-hoc test showed a faster reaction time after the intervention as compared to the pre-intervention only in the active tDCS group (*t* = 7.28, *p* < 0.001). Here, however, mean reaction time at baseline was significantly different across groups, but not after intervention (Fig. 4M-P).

### Interplay between cognitive improvements and mental health outcomes

**Fig. 5:**
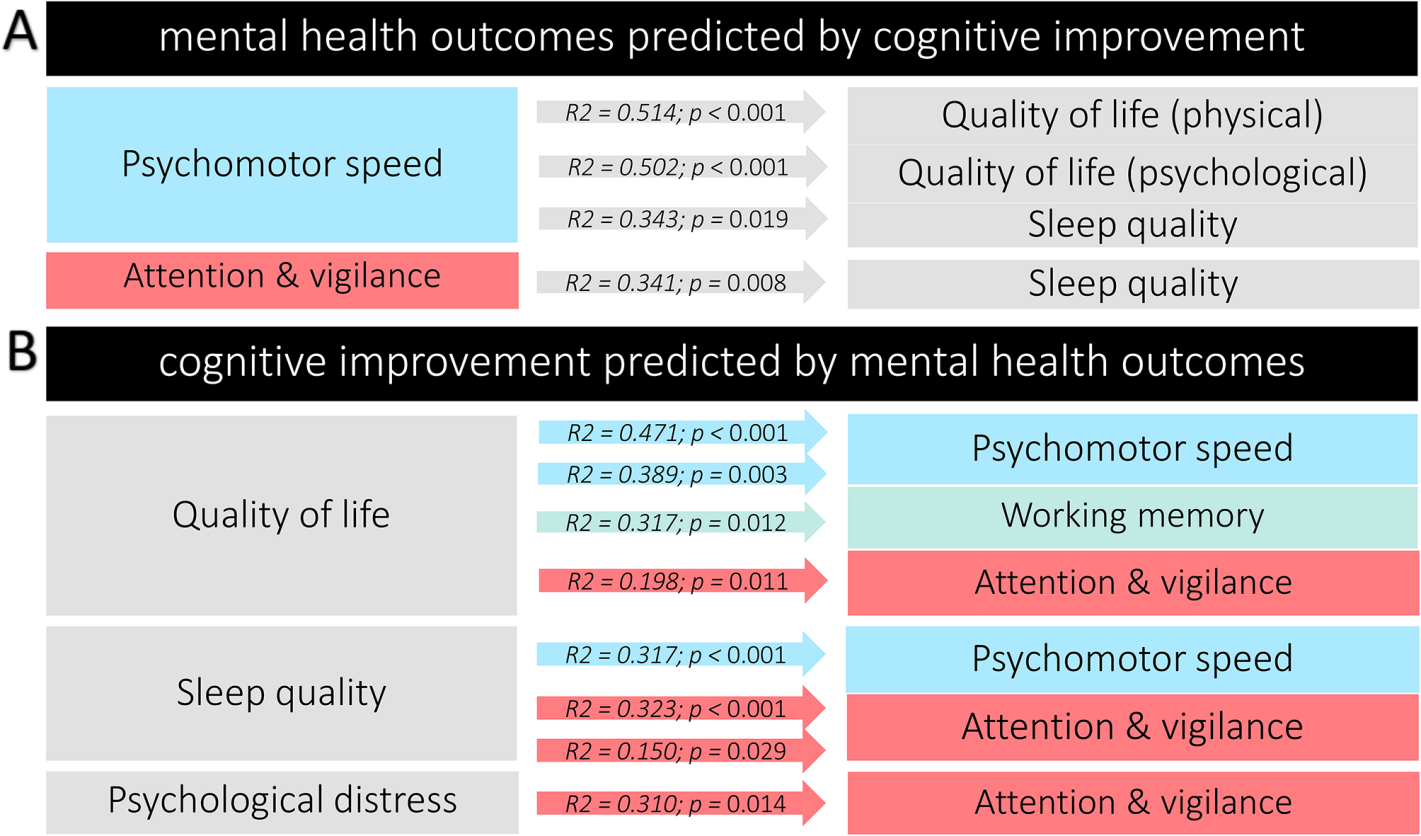
The relationship between cognitive improvement and mental health outcomes. (**A**) Results of multiple linear regressions predicting how enhanced cognitive performance forecasts improved mental health in MS patients. (**B**) Results of multiple linear regressions predicting how improved mental health forecasts enhanced cognitive performance in MS patients

To investigate the relationship between cognitive functions and mental health outcomes, we performed a correlational analysis followed by linear regression analyses. We first calculated Pearson’s correlations between mental health outcomes and cognitive task performance after intervention. Enhanced quality of life was significantly correlated with faster performance in the psychomotor task (*r*_5choice − move−time_=-0.383, *p* < 0.05, *r*_5choice − reaction−time_=-0.481, *p* < 0.01, *r*_simple−move−time_=-0.587, *p* < 0.01) and improved attention/vigilance (i.e., higher performance accuracy) also showed negative correlations with insomnia (*r*=-0.434, *p* < 0.05), hypersomnia (*r*=-0.423, *p* < 0.05), and overall sleep difficulties (*r*=-0.478, *p* < 0.01) (i.e., higher accuracy was associated with lower sleep disturbances). Other significant correlations are detailed in supplementary Table S2. Following correlational analysis, multiple regression analyses were conducted to see how changes in cognitive performance predict mental health outcome variables and vice versa. Briefly, improvements in psychomotor speed and attention/vigilance after the intervention significantly predicted better quality of life and sleep quality. In turn, a higher quality of life predicted cognitive improvements across all three domains, while attention and vigilance were also influenced by sleep quality and psychological distress (Fig. 5, supplementary Table S3). Due to the relatively low sample size, these findings should be interpreted with caution.

## Discussion

As a neuroinflammatory disease, MS impairs neuronal efficacy and neuroplasticity. Non-pharmacological approaches, such as non-invasive brain stimulation, have the potential to enhance the outcomes of pharmacological and physical interventions in MS by enhancing neuroplasticity and functional connectivity [45–47] and potentially have anti-inflammatory effects as observed in several NIBS studies [48, 49], although the latter is not completely clarified. In this randomized, double-blind study, we investigated the effectiveness of repeated prefrontal tDCS in enhancing mental health and cognitive functions in MS patients to assess their impact and potential associations between improvements. Patients receiving real tDCS reported a significantly better quality of life and fewer sleep issues post-intervention, along with lower psychological distress levels compared to the sham group. Additionally, those treated with prefrontal tDCS outperformed in a neuropsychological test battery assessing psychomotor speed, working memory, and visual attention, areas often compromised in MS patients.

The use of tDCS to improve motor and/or cognitive deficits in MS has been less common compared to other neurological/psychiatric conditions. Moreover, results have been mixed so far. While some studies showed promising benefits in ameliorating fatigue, pain, and cognitive symptoms, but inconsistent effects of tDCS on motor symptoms [25, 26], others show a more promising effect on motor functions [27] which could partially be due to heterogeneous protocols applied in these studies, but also different patient characteristics. This is the first study that specifically investigated the efficacy of tDCS on quality of life, mental health-related variables and cognitive functioning of patients with MS. Our study provides supportive evidence for the efficacy of tDCS in improving quality of life and amelioration of psychological and cognitive deficits in patients with MS. Here, the applied protocol and proposed mechanisms of effects are discussed.

### Prefrontal tDCS for promoting mental health and improving cognition in MS

Prefrontal NIBS, including tDCS, have been widely used to enhance mental health and cognitive functions in clinical populations [50–52]. In the same line, our findings show that the intervention improved patients’ quality of life and reduced sleep difficulties, depression, and anxiety. Sleep improvement after the active tDCS can be explained by the more recently stressed role of the cortico-thalamo-cortical feedback loop, which is a top-down regulatory system related to cortical areas (e.g., PFC), in regulating arousal and sleep [53]. Mood-alleviating effects can be partially explained by enhanced cognitive control as a result of tDCS over the DLPFC, which regulates mood and valence of emotional experiences [54, 55]. Importantly, there is a link between the prefrontal cortex, especially the DLPFC, the medial PFC, and the amygdala network in MS patients who experience emotional difficulties [56], and reduced depression, anxiety, and distress following intervention might be explained via this prefrontal-amygdala related emotion regulation. Briefly, upregulating DLPFC activity can reduce amygdala activity [57], which is usually hyperactive during negative emotional processing in MS [56]. This effect, coupled with the functional connectivity-enhancing effect of tDCS [58], may be a possible mechanism of action.

In addition to mental health-related variables, our intervention aimed to improve cognitive deficits in MS patients. The rationale behind the use of prefrontal tDCS for enhancing cognition mostly comes from the involvement of different regions of the prefrontal cortex in various aspects of cognitive functions [52, 59, 60] which seems to be at least partially applicable for MS. Cognitive impairment in MS is the consequence of widespread lesions in the brain and frequently includes deficits in complex attention, efficiency of information processing, executive functioning, processing speed, and long-term memory [4, 18]. A recent large-scale neuroimaging study demonstrated a causal link between MS pathophysiology and various brain regions, including the frontal and prefrontal cortices—particularly the orbitofrontal cortex—and connected subcortical areas like the parahippocampal gyrus [23]. Our study applied tDCS over DLPFC and right FPC which are important for cognitive deficits in MS and improved psychomotor speed and attention/vigilance. These findings align with extensive evidence supporting pro-cognitive effects of prefrontal tDCS in neuropsychiatric disorders marked by executive and cognitive deficits, including mood and anxiety disorders [61, 62], obsessive-compulsive disorder [63, 64], schizophrenia [65, 66], and substance use disorder [67, 68]. Pro-cognitive effects have also been observed in neurological disorders like Parkinson’s disease and stroke [69, 70] as well as in major neurodevelopmental disorders such as ADHD and autism [71–73].

Comparing our study’s findings with previous tDCS studies in MS is also informative. Recent systematic reviews and metanalyses of tDCS studies conducted in MS show inconsistent results about the cognitive effects of tDCS with some studies reporting clear benefits in ameliorating cognitive symptoms [25], some partial improvement in specific aspects of cognition (e.g., vigilance) [27] and some with no strong evidence for effectiveness of tDCS on cognition [26]. Studies with a focus on cognitive functions have typically applied anodal tDCS to the left DLPFC, using return electrodes on the right DLPFC, right shoulder, or right supraorbital area, with stimulation intensities ranging from 1.5 to 2 mA and durations between 20 and 30 min [27]. Our study aimed to shed light on the effects of tDCS on specific secondary deficits, but also on the interplay between cognitive functions and mental health domains, both significantly affected by MS but understudied in previous research. Importantly, here we observed an interplay between mental health outcomes and cognitive improvement, indicating that the impaired domains of mental health and cognitive deficits are interrelated in MS.

One consideration regarding the protocol in the present study (anodal F3- cathodal Fp2) is the use of the reference electrode over the right FPC instead of the right DLPFC. The bilateral DLPFC protocol can also be applied if cognitive functions are the main focus, as indicated in previous tDCS studies in MS [27] and other neuropsychiatric disorders [50, 74]. In this study, we also aimed to explore the impact of the intervention on participants’ emotional experiences. FPC, which includes the orbitofrontal cortex [41], was selected over the right DLPFC due to its significant role in emotional regulation and direct connections with subcortical regions [59]. The relative efficacy of DLPFC-FPC versus bilateral DLPFC tDCS needs to be compared to determine which is more effective in enhancing mental health and cognition-related variables. In this line, a more comprehensive understanding of tDCS mechanisms can be achieved by incorporating neurophysiological measures that assess changes in cortical excitability (e.g., TMS paradigms) [44], functional connectivity (e.g., EEG, fMRI), and biomarkers related to inflammation (e.g., BDNF) and neuroplasticity (e.g., EEG, TMS-EEG) [75]. This data can inform personalized treatment strategies and enhance therapeutic outcomes for MS patients.

### tDCS broader application for mental health improvement in MS

In addition to improving cognitive and mental health outcomes for MS patients, tDCS can address a broader range of emotional difficulties in more practical and feasible contexts. It can specifically address mood dysregulation and fatigue— two common issues in MS—making them priority areas for tDCS and NIBS interventions. A large body of tDCS studies have shown that consecutive applications of tDCS can improve depression [76–78] and fatigue [79, 80] suggesting their use for promoting mental health in MS patients. A key advantage of tDCS for these issues is its suitability for home use in contrast to modalities like transcranial magnetic stimulation. Recent advancements in home-based applications of transcranial electrical and alternating current stimulation support the safety and feasibility [81, 82] of tele-supervised home-based tDCS for major depressive disorder [77, 83], Alzheimer’s disease [84], and fatigue in MS patients [85]. This can have clinical implications for future applications of tDCS in MS.

### Limitations

We acknowledge that our study has some limitations. Firstly, although the sample size of this study is larger than the majority of the previous tDCS studies in MS [26, 27], it is still relatively small, and therefore, these findings need to be confirmed in larger trials in the future. Secondly, we did not measure outcome variables over a reasonable follow-up period to determine how long the observed effects lasted and the intervention was not applied daily but every second or third day, which might have limited efficacy. The double-blind procedure should be implemented with allocation concealment and devices featuring sham features for the experimenters, which was not feasible in this study. Finally, we did not include any physiological measures, which would have been informative to understand how brain functions and physiology are affected by the intervention and how they align with the behavioural results.

## Conclusions

In conclusion, this study investigated how anodal and cathodal tDCS targeting the left DLPFC and right FPC affect quality of life, sleep difficulties, psychological distress, and cognitive functions in MS patients after 10 brain stimulation sessions. The group that received real intervention vs. the placebo group, showed significant improvement in quality of life, and reduced psychological distress and sleep difficulties. These enhancements correlated with increased psychomotor speed and attention/vigilance. Improvements in cognitive function and mental health outcomes were interrelated. These findings suggest that repeated DLPFC-FPC tDCS has the potential to be a safe and promising intervention for treating secondary symptoms (mental-health-related variables and cognitive deficits) in patients with MS. Future studies are recommended to include neurophysiological measures alongside behavioral outcomes to enhance understanding of tDCS mechanisms in MS and to provide a more comprehensive evaluation of tDCS efficacy.

## Electronic supplementary material

Below is the link to the electronic supplementary material.

Supplementary Material 1

## Data Availability

The datasets used and/or analyzed during the current study are publicly available: https://figshare.com/articles/dataset/Mental_Health_Cognitive_Function_Data/26490208.

## Abbreviations

MS: Multiple Sclerosis
tDCS: transcranial direct current stimulation
MSIS-29: Multiple Sclerosis Impact Scale
MSQ: Mini Sleep Questionnaire
DASS-21: Depression Anxiety Stress Scale-21
RTI: Reaction Time task
SWM: Spatial Working Memory task
RVP: Rapid Visual Information Processing task
DLPFC: Dorsolateral prefrontal cortex
FPC: Frontopolar cortex
