# Supplementary material for: Repeated prefrontal tDCS for improving mental health and cognitive deficits in multiple sclerosis: a randomized, double-blind, parallel-group study
Source: J Transl Med. 2024 Sep 13;22:843. doi: 10.1186/s12967-024-05638-1 (PMC11397099; doi:10.1186/s12967-024-05638-1)
Supplement: Supplementary file 1 — Supplementary Material 1 [file 12967_2024_5638_MOESM1_ESM.docx]

- 1. **Measures**
  2. ***Mental health-related measures***
     1. *Multiple Sclerosis Impact Scale (MSIS-29)*

The Multiple Sclerosis Impact Scale (MSIS-29) serves as an essential tool in evaluating quality of life within medical research and clinical practice contexts (Hobart et al., 2001). This scale consists of 29 items, with the first 20 concentrating on physical aspects and the remaining 9 targeting psychological aspects. Participants are required to rate each question using a Likert-type scale ranging from 1 (not at all) to 5 (extremely). Rosti-Otajärvi et al. (2017) conducted a study that found that the data quality for the MSIS-29 was exceptional. They reported that 99.5% of scores for the physical scale and 99.3% for the psychological scale were computable. The study also revealed that internal consistency was very high, with Cronbach's alpha values of 0.97 for the physical subscale and 0.90 for the psychological subscale. Additionally, the physical subscale demonstrated strong correlations with physical functioning measures, including disease severity and the quality of life's mobility domain. Similarly, the psychological subscale exhibited the highest correlations with self-reported fatigue and the quality of life's anxiety/depression domains(Rosti-Otajärvi et al., 2017) Furthermore, Ayatollah et al. (2006) reported good internal consistency (0.75) and reliability (intraclass correlation coefficients 0.70) for the Iranian version of MSIS-29(Ayatollahi et al., 2006). In our sample, the Cronbach's alpha values for the physical and mental scales were 0.94 and 0.91, respectively.

- - 1. *Mini Sleep Questionnaire (MSQ)*

The mini sleep questionnaire consists of six questions that participants answer on a 5-point Likert scale, ranging from "very little" (1 point) to "very much" (5 points). Three questions are related to hypersomnolence, and three are associated with sleep insomnia. A high score on this questionnaire indicates excessive sleepiness, while a low score indicates insomnia(Hosseini et al., 2020). The criterion validity was reported as favorable, and a reliability of 0.90 was reported . To evaluate the reliability of the sleep disorders questionnaire in the Iranian population, the Cronbach’s alpha test was conducted, resulting in a score of 0.79. To assess validity, exploratory factor analysis was undertaken, revealing that the scale accounted for 61% of the variance in scores of questions about sleep disorders (Manavipour, 2015). In our sample, Cronbach's alpha was 0.68, 0.45, and 0.67 for sleep quality, insomnia, and hypersomnia, respectively***.***

- - 1. *Depression Anxiety Stress Scale-21 (DASS-21)*

The Depression, Anxiety, and Stress Scale-21 (DASS-21) is a standardized self-report questionnaire designed to measure the severity of depression, anxiety, and stress symptoms in individuals without prior diagnoses. Each item is scored on a four-point Likert scale ranging from 0 to 3, with subscale scores needing to be doubled for the final result. Studies have reported strong internal consistency for the DASS-21, with Cronbach's alpha values of 0.94 for depression and 0.87 for anxiety, as well as 0.92 for stress and 0.81 for anxiety. Sahebi et al. (2005) conducted a study to evaluate the validity of the Iranian version of the DASS-21. The study employed factor analysis and criterion validity (concurrent method) for measurement. The findings revealed a correlation of +0.70 between the Depression subscale and the Beck Depression Inventory scale, a correlation of +0.67 between the Anxiety subscale and Zung Anxiety Inventory, and a correlation of +0.49 between the Stress subscale and Perceived Stress Inventory, indicating its reliability for the Iranian population(Karimi et al., 2020). Furthermore, Kakemam et al. (2022) reported acceptable Cronbach's alpha values of 0.79, 0.91, and 0.93 for anxiety, stress, and depression, respectively, along with acceptable test-retest reliability (0.740-0.881, P < 0.01) for DASS-21 and its dimensions(Kakemam et al., 2022). In the present sample, Cronbach's alpha values of 0.89, 0.81, and 0.78 were found for depression, anxiety, and stress subscales, respectively, indicating good reliability***.***

- 1. ***Neuropsychological battery***
     1. *Reaction Time (RTI)*

RTI is a measure of psychomotor speed and attention (Langley et al., 2023). During the task, the participant selects and holds a button at the bottom of the screen. Circles are presented above (one for the simple mode, and five for the five-choice mode). In each case, a yellow dot appears in one of the circles, and the participant must react quickly, releasing the button at the bottom of the screen and selecting the circle with the dot. In RTI different outcome variables are yielded. We were interested in the simple and 5-choice movement and reaction times. RTI Simple movement time (RTI SMT): This is the time required to touch the stimulus on the screen after the pad button has been released in trials where the stimuli appear in only one location. A lower score is better. RTI Simple reaction time (RTI SRT): This is the speed with which the participant releases the push button in response to the initiation of a stimulus at a single location; reaction time latency is measured in milliseconds. A smaller value is better. There are

- - 1. *Spatial Working Memory (SWM)*

The SWM task starts with several colored squares (boxes) displayed on the screen. The objective of this task is for the participant to identify one yellow 'token' in each of the boxes by employing a process of elimination and then utilizing them to populate an empty column on the right side of the screen. The number of boxes can be incrementally raised based on the test's difficulty level, with a maximum of 12 boxes shown for participants to examine. To prevent the use of stereotyped search techniques, the color and location of the boxes are varied from one trial to another. We were interested in the strategy scores and errors. SWM Total errors (SWM TE): This is the number of times a box is selected that certainly does not contain a blue token and therefore should not have been visited by the subject. Lower is better. SWM Strategy: An estimate of the use of this strategy is obtained by counting the number of times the participant starts a new search with a different box only for six and eight-box problems. A high score represents poor use of strategy, and a low score corresponds to effective use.

- - 1. *Rapid Visual Information Processing (RVP)*

The RVP is a sensitive measure of sustained visual attention (Langley et al., 2023) and presents participants with a white box in the center of the computer screen, inside which digits from 2 to 9 appear in a pseudo-random order at the rate of 100 digits per minute. Participants are asked to detect target sequences of digits and register responses using the press pad. It takes about 10 min to complete and the output measure includes latency which is a good indicator of sustained attentional function.

**Table S1**: Results of the mixed model ANOVAs for effects of group (active tDCS, sham) and time (pre-intervention, post-intervention) on outcome measures.

| **Measure** | **Outcome variable** | **Source** | **df** | **F** | ***p*-value** | **partial eta^2^** |
| --- | --- | --- | --- | --- | --- | --- |
| (MSIS) Quality of life | score | Time | 1, 30 | 7.945 | 0.008 | 0.209 |
|  |  | Group | 1, 30 | 0.949 | 0.338 | 0.031 |
|  |  | time×group | 1, 30 | 9.987 | 0.004 | 0.250 |
| DASS | score | Time | 1, 30 | 6.13 | 0.019 | 0.170 |
|  |  | Group | 1, 30 | 3.877 | 0.058 | 0.114 |
|  |  | Domain | 2, 60 | 15.608 | **<0.001** | 0.342 |
|  |  | time×group | 1, 30 | 23.505 | **<0.001** | 0.439 |
|  |  | time× domain | 2, 60 | 0.243 | 0.785 | 0.008 |
|  |  | group× domain | 2, 60 | 1.156 | 0.322 | 0.037 |
|  |  | time×group× domain | 2, 60 | 1.137 | 0.328 | 0.037 |
| Sleep Total score | score | Time | 1, 30 | 30.598 | **<0.001** | 0.505 |
|  |  | Group | 1, 30 | 1.065 | 0.310 | 0.034 |
|  |  | time×group | 1, 30 | 37.314 | **<0.001** | 0.554 |
| Insomnia | score | Time | 1, 30 | 19.756 | **<0.001** | 0.397 |
|  |  | Group | 1, 30 | 0.277 | 0.603 | 0.009 |
|  |  | time×group | 1, 30 | 25.004 | **<0.001** | 0.455 |
| Oversleep | score | Time | 1, 30 | 21.861 | **<0.001** | 0.422 |
|  |  | Group | 1, 30 | 1.265 | 0.270 | 0.04 |
|  |  | time×group | 1, 30 | 25.667 | **<0.001** | 0.461 |
| 5 choice movement time | score | Time | 1, 35 | 127.32 | **<0.001** | 0.784 |
|  |  | Group | 1, 35 | 2.412 | 0.129 | 0.064 |
|  |  | time×group | 1, 35 | 73.491 | **<0.001** | 0.677 |
| 5 choice reaction time | score | Time | 1, 35 | 11.684 | **0.002** | 0.25 |
|  |  | Group | 1, 35 | 1.299 | 0.262 | 0.036 |
|  |  | time×group | 1, 35 | 34.992 | **<0.001** | 0.50 |
| simple movement time | score | Time | 1, 35 | 17.476 | **<0.001** | 0.333 |
|  |  | Group | 1, 35 | 0.844 | 0.365 | 0.024 |
|  |  | time×group | 1, 35 | 20.905 | **<0.001** | 0.374 |
| simple reaction time | score | Time | 1, 35 | 24.729 | **<0.001** | 0.414 |
|  |  | Group | 1, 35 | 6.439 | **0.016** | 0.155 |
|  |  | time×group | 1, 35 | 40.317 | **<0.001** | 0.535 |
| SWM strategy | score | Time | 1, 35 | 0.703 | 0.740 | 0.003 |
|  |  | Group | 1, 35 | 0.493 | 0.367 | 0.023 |
|  |  | time×group | 1, 35 | 0.823 | 0.208 | 0.045 |
| SWM total error | score | Time | 1, 35 | 9.081 | **0.005** | 0.206 |
|  |  | Group | 1, 35 | 0.005 | 0.946 | 0.001 |
|  |  | time×group | 1, 35 | 1.778 | 0.191 | 0.048 |
| RVP Hits | score | Time | 1, 35 | 17.797 | **<0.001** | 0.337 |
|  |  | Group | 1, 35 | 2.535 | 0.120 | 0.048 |
|  |  | time×group | 1, 35 | 46.299 | **<0.001** | 0.569 |
| RVP mean latency | score | Time | 1, 35 | 211.259 | **<0.001** | 0.858 |
|  |  | Group | 1, 35 | 30.052 | **<0.001** | 0.462 |
|  |  | time×group | 1, 35 | 241.504 | **<0.001** | 0.873 |

*Note*: tDCS = transcranial Direct Current Stimulation; M = Mean; SD = Standard Deviation; MSIS= Multiple Sclerosis Impact Scale(quality of life); DASS= Depression, anxiety, stress scale; MSQ= Mini sleep questionnaire(total sleep score, insomnia and oversleep); RTI= Reaction time test(RTI Mean simple reaction time, RTI Mean five-choice reaction time, RTI Mean simple movement time, and RTI Mean five choice movement time); SWM= Spatial Working Memory(SWM Strategy, SWM Total errors, and SWM Between); RVP= Visual Information Processing (RVP Hits, RVP Miss, RVP mean latency); Significant results are highlighted (*p* ≤ 0.05) in **bold**.

Table S2: Correlations of mental health-related and cognitive variables

|  | 1 | 2 | 3 | 4 | 5 | 6 | 7 | 8 | 9 | 10 | 11 | 12 | 13 | 14 |
| --- | --- | --- | --- | --- | --- | --- | --- | --- | --- | --- | --- | --- | --- | --- |
| (MSIS) Quality of life | 1 |  |  |  |  |  |  |  |  |  |  |  |  |  |
| oversleep | -0/247 | 1 |  |  |  |  |  |  |  |  |  |  |  |  |
| insomania | -0/281 | .595^**^ | 1 |  |  |  |  |  |  |  |  |  |  |  |
| Sleep total score | -0/290 | .936^**^ | .839^**^ | 1 |  |  |  |  |  |  |  |  |  |  |
| ASWM.Total error | -0/148 | -0/031 | 0/285 | 0/103 | 1 |  |  |  |  |  |  |  |  |  |
| SWM Strategy | -0/157 | -0/116 | 0/238 | 0/026 | .644^**^ | 1 |  |  |  |  |  |  |  |  |
| 5 choice movement time | -0/338 | 0/092 | 0/163 | 0/133 | -0/018 | 0/210 | 1 |  |  |  |  |  |  |  |
| 5 choice reaction time | -.383^*^ | 0/194 | 0/088 | 0/170 | 0/169 | 0/142 | 0/035 | 1 |  |  |  |  |  |  |
| simple movement time | -.481^**^ | 0/031 | 0/186 | 0/102 | 0/185 | 0/151 | 0/340 | 0/346 | 1 |  |  |  |  |  |
| simple reaction time | -.587^**^ | .509^**^ | .395^*^ | .517^**^ | 0/220 | 0/142 | .369^*^ | 0/315 | 0/231 | 1 |  |  |  |  |
| RVP correct rejections | .775^**^ | -0/267 | -.446^*^ | -.375^*^ | -0/208 | -0/262 | -.541^**^ | -.415^*^ | -.524^**^ | -.650^**^ | 1 |  |  |  |
| RVP false alarms | -0/345 | 0/245 | .436^*^ | .356^*^ | 0/275 | 0/316 | .547^**^ | 0/166 | 0/313 | .418^*^ | -.681^**^ | 1 |  |  |
| RVP Hits | 0/270 | -.434^*^ | -.423^*^ | -.478^**^ | 0/025 | 0/137 | -0/180 | 0/140 | 0/169 | -.430^*^ | .368^*^ | -.441^*^ | 1 |  |
| RVP mean latency | 0/042 | -0/292 | -0/080 | -0/232 | 0/074 | 0/176 | 0/012 | 0/181 | 0/261 | -0/133 | 0/177 | -0/278 | .432^*^ | 1 |

*Note* MSIS= Multiple Sclerosis Impact Scale (quality of life); SWM= Spatial Working Memory; RTI= Reaction time test; RVP= Visual Information Processing; **. Correlation is significant at the 0.01 level (2-tailed); *. Correlation is significant at the 0.05 level (2-tailed).

**Table S3**: Results of multiple regression analyses.

| **Dependent variable** | **Measure** | **Predictor** | **R^2^** | **df** | ***F*_model_** | ***p*-value** |
| --- | --- | --- | --- | --- | --- | --- |
| **COGNITION variables as predictors** | | | | | | |
| Quality of life | Physical | Psychomotor speed | 0.514 | 4,27 | 7.142 | **<0.001** |
|  | Psychological | Psychomotor speed | 0.502 | 4,27 | 6.795 | **<0.001** |
| Sleep Quality |  | Psychomotor speed | 0.343 | 4,27 | 3.529 | **0.019** |
|  |  | Attention & vigilance | 0.341 | 3,28 | 4.829 | **0.008** |
| **MENTAL HEALTH variables as predictors** | | | | | | |
| Psychomotor speed | 5 choise Movement RT | Quality of life | 0.471 | 3,28 | 8.313 | **<0.001** |
|  | Simple Movement RT | Quality of life | 0.389 | 3,28 | 5.934 | **0.003** |
|  | Simple RT | Quality of life | 0.412 | 3,28 | 2.540 | **0.077** |
|  | Simple RT | Sleep Quality | 0.317 | 1,30 | 13.919 | **0.001** |
| Attention & vigilance |  | Sleep Quality | 0.323 | 1,30 | 14.331 | **0.001** |
|  |  | Quality of life | 0.092 | 3,28 | 0.945 | 0.432 |
|  |  | Psychological distress | 0.310 | 3,28 | 4.202 | **0.014** |
| Working memory | Strategy | Quality of life | 0.317 | 3,28 | 3.341 | **0.012** |

Significant results are highlighted (*p* ≤ 0.05) in **bold**.

**References**

Ayatollahi, P., Nafissi, S., Eshraghian, M., & Tarazi, A. (2006). Cross-Cultural Adaptation Of The Multiple Sclerosis Impact Scale (MSIS-29) For Iranian MS Patients, Evaluation Of Reliability And Validity. *Tehran-Univ-Med-J*, *64*(1), 62-68. <http://tumj.tums.ac.ir/article-1-1006-fa.html>

Hobart, J., Lamping, D., Fitzpatrick, R., Riazi, A., & Thompson, A. (2001). The Multiple Sclerosis Impact Scale (MSIS-29): a new patient-based outcome measure. *Brain: A Journal of Neurology*, *124*(Pt 5), 962-973. <https://doi.org/10.1093/brain/124.5.962>

Hosseini, S. M., Seddighi, A. S., Seddighi, A., & Nikouei, A. (2020). Validity and Reliability of the Mini Sleep Questionnaire-Persian Version (MSQ-P). *Journal of Sleep Disorders & Therapy*, *9*(5). <https://doi.org/10.35248/2167-0277.20.9.317>

10.35248/2167-0277.20.9.317.Copyright

Kakemam, E., Navvabi, E., Albelbeisi, A. H., Saeedikia, F., Rouhi, A., & Majidi, S. (2022). Psychometric properties of the Persian version of Depression Anxiety Stress Scale-21 Items (DASS-21) in a sample of health professionals: a cross-sectional study. *BMC Health Serv Res*, *22*(1), 111. <https://doi.org/10.1186/s12913-022-07514-4>

Karimi, S., Andayeshgar, B., & Khatony, A. (2020). Prevalence of anxiety, depression, and stress in patients with multiple sclerosis in Kermanshah-Iran: a cross-sectional study. *BMC Psychiatry*, *20*, 166. <https://doi.org/10.1186/s12888-020-02579-z>

Langley, C., Sahakian, B. J., & Robbins, T. W. (2023). Cambridge Neuropsychological Test Automated Battery (CANTAB). *The SAGE Handbook of Clinical Neuropsychology: Clinical Neuropsychological Assessment and Diagnosis*, 435.

Manavipour, D. (2015). Psychometric Properties of the Sleep Questionnaire. *The-Neuroscience-Journal-of-Shefaye-Khatam*, *3*(3), 15-20. <https://doi.org/10.18869/acadpub.shefa.3.3.15>

Rosti-Otajärvi, E., Hämäläinen, P., Wiksten, A., Hakkarainen, T., & Ruutiainen, J. (2017). Validity and reliability of the Finnish version of the Multiple Sclerosis Impact Scale-29. *Brain and Behavior*, *7*(7), e00725. <https://doi.org/10.1002/brb3.725>
